# Glycated albumin: a potential biomarker in diabetes

**DOI:** 10.1590/2359-3997000000272

**Published:** 2022-05-01

**Authors:** Priscila Aparecida Correa Freitas, Lethicia Rozales Ehlert, Joíza Lins Camargo

**Affiliations:** 1 Universidade Federal do Rio Grande do Sul Porto Alegre RS Brasil Programa de Pós-graduação em Ciências Médicas – Endocrinologia, Universidade Federal do Rio Grande do Sul (UFRGS), Porto Alegre, RS, Brasil; 2 Hospital de Clínicas de Porto Alegre Porto Alegre RS Brasil Serviço de Endocrinologia, Hospital de Clínicas de Porto Alegre (HCPA), Porto Alegre, RS, Brasil

**Keywords:** Diabetes mellitus, glycated albumin, glycated proteins

## Abstract

Diabetes mellitus (DM) is a chronic and metabolic disease that presents a high global incidence. Glycated hemoglobin (A1C) is the reference test for long-term glucose monitoring, and it exhibits an association with diabetic chronic complications. However, A1C is not recommended in clinical situations which may interfere with the metabolism of hemoglobin, such as in hemolytic, secondary or iron deficiency anemia, hemoglobinopathies, pregnancy, and uremia. The glycated albumin (GA) is a test that reflects short-term glycemia and is not influenced by situations that falsely alter A1C levels. GA is the higher glycated portion of fructosamine. It is measured by a standardized enzymatic methodology, easy and fast to perform. These laboratory characteristics have ensured the highlight of GA in studies from the last decade, as a marker of monitoring and screening for DM, as well as a predictor of long-term outcomes of the disease. The aim of this review was to discuss the physiological and biochemistry characteristics of the GA, as well as its clinical utility in DM.

## INTRODUCTION

Diabetes mellitus (DM) is a chronic metabolic disease caused by diminished or absent secretion of insulin or even by reduced tissue sensitivity to insulin (1,2). Presently, DM is a worldwide epidemic and a great challenge to health care systems everywhere. The International Diabetes Federation (IDF) estimates that one in eleven adults have DM, totalizing approximately 415 million people, and 193 million of them have not yet been diagnosed (1).

Chronic hyperglycemia is a common feature in all subtypes of this disease and is associated with long-term damage, which increases the morbidity and mortality rates and causes dysfunction of different organs, such as kidney failure, blindness, and amputation of limbs (2). These chronic complications are costly to the health care systems and reduce the life expectancy of diabetic patients (1,2).

Currently, the laboratory tests used to diagnose DM are glycated hemoglobin (A1C), fasting plasma glucose (FG) and two-hour plasma glucose (2hG) after a 75g oral glucose tolerance test (OGTT) (2,3). A1C is also the reference test for glycemic monitoring since it directly reflects mean glycemia (5) and is strongly correlated to the long-term complications of DM (4,5). However, the use of A1C is not recommended in some clinical situations that influence the hemoglobin metabolism (3,6,7), due to the possible interference in its results making them misinterpreted. Moreover, recent studies have shown a disagreement between the A1C levels in different ethnic groups for equal levels of glycemia (8,9), but the reasons for these disparities have not yet been well explained.

Glycated albumin (GA) is a laboratory test that has gained some importance for glycemic monitoring in DM in the last decades (10,11). GA is one of the fructosamines, but it has the advantage of not being influenced by the concentration of other serum proteins since it is specific to the albumin glycation rates (12). Further, GA does not require fasting for its measurement and reflects short-term glycemia due to the half life time of the albumin, which is approximately 3 weeks. Compared to A1C, GA is not affected by the presence of hemolytic processes and abnormal Hb (13). Besides, in conditions such as anemia, pregnancy, postprandial hyperglycemia and DM using insulin, GA seems to be a better glycemic marker than A1C (11) and also it is especially indicated for diabetic patients on hemodialysis (14,15). Recently, studies on type 1 (16) and type 2 DM patients (17) reported an association of GA with the chronic complications of the disease.

Although GA is being studied in the last few years, this test is not yet widely used in laboratory routine, and few commercial reagents are available on the market to its analysis. However, the results of clinical investigations make GA a promising marker in DM. In this context, the proposal of this review is to present the physiological and laboratory characteristics of GA, and discuss its clinical usefulness in the diagnosis and management of DM.

## BIOCHEMICAL CHARACTERISTICS OF GA AND BIOLOGICAL IMPACT OF GLYCATION

Albumin is a high molecular weight protein with 66.7 kDa, composed of a single polypeptide chain which contains 585 amino acids, 17 disulfide bridges and 3 homologous domains that are connected in a helical structure (18). It is the main plasma protein, representing about 60% of the total proteins in the blood, with concentrations between 3.0 and 5.0 g/dL and a half-life of 14 to 20 days (18,19). Albumin structure makes it easier to perform its physiological functions, such as maintaining pH and blood osmotic pressure. Also, albumin acts as a powerful antioxidant and as the main transporter of metabolic products, ions, nutrients, drugs, hormones and fatty acids (20).

**Figure 1:**
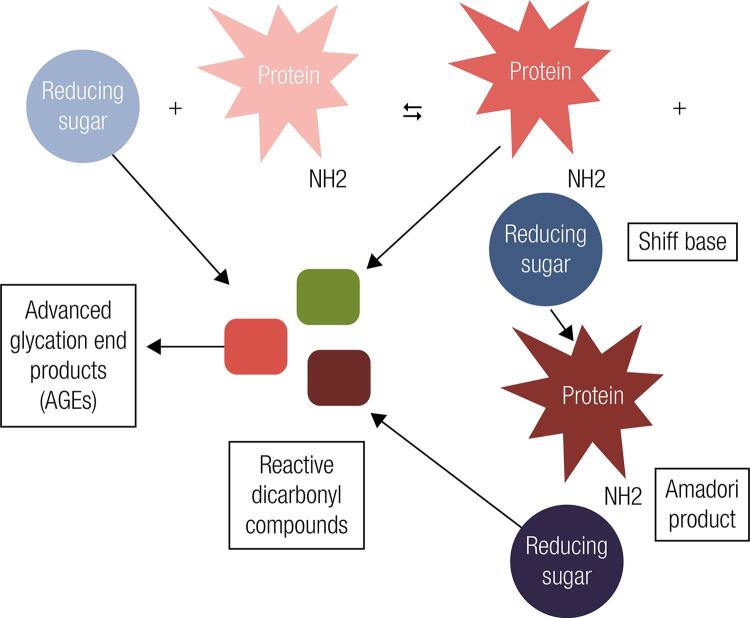
Maillard reaction illustration. In the first glycation stage, there is production of Shiff base by a reaction between a reducing sugar and a free amine group present into the polypeptide chain of plasma proteins and, subsequently, a rearrangement yield the Amadori product. In the following stages, the degradation of the Shiff base and Amadori products, as well as sugar autoxidation are responsible for forming reactive dicarbonyl compounds, known as AGEs’ precursors.

Similar to the other proteins, albumin also goes through the physiologic process of glycation (21). By definition, glycation is a non-enzymatic spontaneous reaction in which a reducing sugar is added to a free amino group, typically lysine or arginine present within proteins, also called as Maillard reaction (Figure 1) (18-20). The first step of this reaction involves the formation of an unstable and reversible product known as Schiff base, formed by the bonding of a carbonyl group of an acyclic carbohydrate with the N-terminal amino acid (19). This intermediate product can suffer a change in its conformation and result in a stable and irreversible ketamine, known as the Amadori product (22). The main Amadori adduct formed is fructoselysine, a reaction between glucose and lysine, which may occur on 59 lysine sites present in albumin (18). However, lysine 525 has been identified as the largest albumin glycation site, which is evidenced both *in vivo* and *in vitro* experiments (23,24). The set of ketamines formed by non-enzymatic glycation of proteins is chemically called “fructosamine”. Among the serum fructosamines, GA is the main constituent, representing about 80% of the total of glycations in plasma (18).

The glucose concentration and time of exposure between protein and sugar are the determining factors for the glycations performed during the life of the protein. In other words, glycation depends on the degree and duration of hyperglycemia (22). Extracellular proteins, such as albumin, may be more susceptible to Amadori rearrangements than intracellular proteins as Hb (18). This is due to plasmatic proteins being directly exposed to plasma glucose. These features could justify the differences in the rates of albumin glycation that are about 9 to 10 times greater than those of hemoglobin (25). However, in the *in vitro* experiment by Ueda and Matsumoto, it was evidenced that GA production was about 4.5 times greater than A1C after adding known and equal concentrations of glucose in previously treated samples from healthy volunteers. These findings showed that even in identical *in vitro* glycation conditions, GA is produced faster than A1C (20).

In advanced glycation stages, additional oxidative and irreversible events occur regarding the glycated proteins, producing stable and heterogeneous compounds known as advanced glycation end products (AGEs – Figure 1). Although the formation of AGEs is a normal process, conditions of typical hyperglycemia in patients with DM increase their production rates (26). AGEs receptors are present in cells of different tissues, such as macrophages, muscle, endothelial and glial cells (27). They are expressed as membrane molecules, constituents of immunoglobulin superfamily and act as signal transduction receptors, inducing oxidative stress and starting an inflammatory cascade by activation of the nuclear factor-κB (NF-κB). NF-κB modulates the gene transcription of pro-inflammatory molecules such as interleukins 1, 6 and 8 and tumor necrosis factor-α, and also the vascular cell adhesion molecule-1 and intercellular adhesion molecule-1 (26). As a consequence of this cascade, there is an increased production of reactive oxygen species, which is directly associated with the pathogenesis and long-term complications in DM (21,27). Kisugi and cols. evaluated samples from a patient with DM during one month of hospitalization due to hyperglycemia symptoms and evidenced that the formation of AGEs was drastically reduced with the concomitant reduction of the GA levels (24).

## LABORATORY MEASUREMENT OF GA

Historically, fructosamine has been used in clinical practice when a short-term glycemia evaluation is needed (12,28). However, this test presents low accuracy since it is influenced by all plasma proteins and also by other molecules present in the blood, such as bilirubin, uric acid and low molecular weight substances (12). Further, fructosamine is not available at all laboratories (18,29) and there are no well-established international standards for its use.

Methods for the evaluation of GA have been developed since the 1980s using serum or plasma samples (28). The older methods presented many disadvantages due to the techniques complexity or the high costs and/or lack of precision. Besides, the non-standardization of these assays corroborated to the unpopularity of GA, and all attention were directed to A1C (30).

GA can be measured by ion-exchange high-performance liquid chromatography (HPLC), boronate affinity chromatography, immunoassays (radioimmunoassay and Enzyme Linked Immuno Sorbent Assay), colorimetric method with thiobarbituric acid and enzymatic methods using proteinase and ketamine oxidase (12,28,29,31), however these methods are currently not available in the laboratory routine (32).

The reference intervals described for GA depend on the method used since GA levels may vary according to the glycation sites analyzed by the assay employed, and also if the method of analysis considers the GA molecule for measurement and not its glycated amino acids (31). For instance, the immunoassay techniques, colorimetric methods with thiobarbituric acid and enzymatic methods consider the glycated amino acids as the reference for the GA levels. On the other hand, the HPLC techniques and other chromatography methods consider the GA molecule to define their levels. Despite this difference, all methods available agree that the proportion of GA in patients with DM increases 2 to 5 fold compared to normoglycemic patients (18).

**Figure 2:**
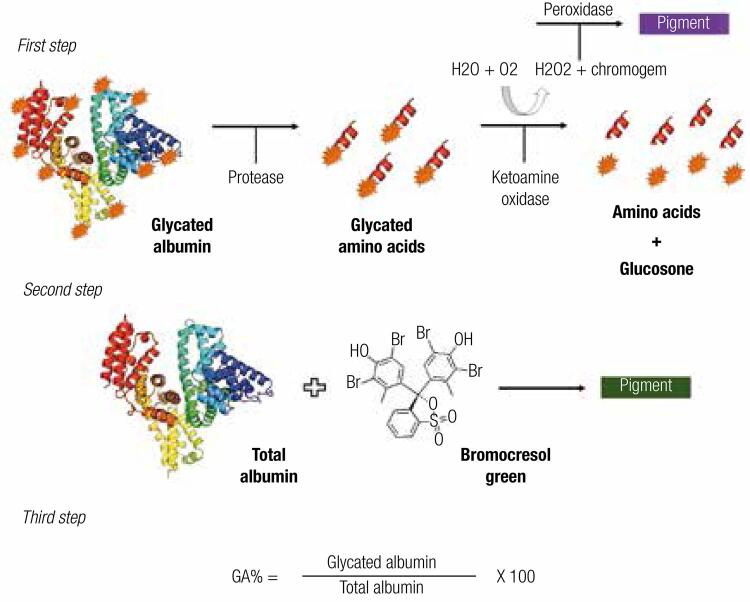
Enzymatic reaction for GA determination. First step: glycated amino acids are released from the GA molecule through an albumin-specific protease. A ketoamine oxidase separates the free amino acids and glucosone, this last an intermediate product of Amadori reaction. The final pigment is proportional to the amount of GA in the sample; Second step: plasma albumin reacts with bromocresol green into an acid environment, resulting in a colored compound that is related to total albumin concentration; Third step: the percentage of GA is obtained by a math calculation considering the two previous reactions.

An enzymatic methodology with a shorter operational time and easier to perform both manually and automatically was proposed to evaluate the GA levels in order to overcome the limitations of the previously existing techniques (12). This method presents three steps (Figure 2), using specific proteinase for albumin and ketamine oxidase, besides the bromocresol green reagent for albumin determination and later calculation of %GA. In the validation performed to introduce the test on the market, the analytic performance was excellent and the assay was not influenced by bilirubin and glucose, but a slight interference in the GA levels in the presence of Hb and ascorbic acid was reported (12). Other studies described similar results, concluding that the new enzymatic methodology, known as “Lucica GA-L^®^” (Asahi Kasei Pharma Corporation, Tokyo, Japan) showed reproducibility, accuracy (31) and a good correlation with A1C (30). Subsequently, other manufacturers have released similarly methodologies for GA analysis, but instead of a specific measurement of its levels, these assays employ math equations to obtain %GA levels (33,34). In addition, the biological variation of GA measured by Lucica GA-L^®^ is lower when compared to fructosamine and A1C (1.7%, 2.8% and, 2.4%, respectively) (35).

GA presents good stability when frozen at very low temperatures. In the study by Kohzuma and cols. frozen samples at -80ºC maintained the GA levels stable for 4 years (31). Watano and cols. found similar results for storage of serum samples at -70ºC. However, they observed a considerable increase in GA levels frozen at -20°C after 6 months (36). Nathan and cols. measured the GA levels in samples of participants in the study The Diabetes Control and Complications Trial Research Group (DCCT) frozen at -70ºC 23 years ago and concluded that the stability of this analyte remained adequate (16).

However, despite all the characteristics cited, the GA test is not yet regularly available in laboratory practice (3), but it has been much used in DM clinical research in the last decade. The factor responsible for the increased number of studies on GA was the consolidation, although without a defined international consensus, of Lucica GA-L^®^ enzymatic assay for GA determination. Even though others enzymatic assays for GA have been launched into the market, currently there are only foreign suppliers available (34). It makes the GA a costlier test than A1C in Brazil. Recently, we compared two different assays for GA and the price per test was around U$ 4 to 6, in contrast with A1C test that is around U$ 2 to 3 in Brazil (34). However, this outlook is likely to change in a near future.

## USE OF GA UNDER CONDITIONS THAT AFFECT A1C

In clinical practice, A1C is used as a reference test for glucose monitoring in DM, and it is also a diagnostic tool (2). However, there are some punctual disadvantages and controversies that limit it use. They are related to certain clinical situations or to the analytical methods employed (3,6). These conditions may yield false results for A1C that are not truly correlated with the mean glycemia (28), and directly affecting the identification and management of patients with DM. In such cases, GA may be an adequate alternative for A1C in the glycemic control (37).

### GA and presence of alterations in Hb

GA can be used as an alternative to A1C in any hematological alteration that interferes in the half-life of red blood cells and/or in the structure or chemical characteristics of Hb (28). Hemolytic anemias and bleeding episodes reduce the A1C values, while iron deficiency anemias, thalassemias, and hemoglobinopathies may elevate its results (6,11,38). During the fetal period, the main type of Hb in the red blood cells is the fetal Hb (HbF), which is gradually replaced by HbA after birth. Since A1C is a glycation product of the HbA, neonates tend to have falsely diminished levels (39). However, the interferences with A1C measurements are method-dependent. Some analytical methodologies may not be affected by common interferences such as hemoglobin variants. The National Glycohemoglobin Standardization Program (NGSP) provides detailed information regarding interference in A1C assays by manufacturers (7).

### GA and pregnancy

During pregnancy, it is recommended that women who already have DM and those who develop gestational DM be followed by glucose self-monitoring and by the A1C levels (2). However, it has been well established that during the last months of pregnancy there is an increased demand for iron, which directly reflects on changes in the A1C throughout the pregnancy (40). In a prospective study by Hashimoto and cols. conducted in pregnant Japanese women with DM, a significant elevation of A1C was found at the end of pregnancy, inversely to the ferritin levels and transferrin saturation. On the other hand, GA remained stable through this period, because it did not suffer interference from the physiological changes characteristic of pregnancy (41).

### GA and chronic kidney disease (CKD)

In patients with DM and CKD, A1C may not be a reliable marker of glycemic control (42). Patients with CKD generally present erythropoietin deficiency and, consequently, they develop anemia. Thus it is necessary to use exogenous erythropoietin to compensate the diminished endogenous synthesis by the kidney, and also iron, which falsely alters the levels of A1C. Further, these patients may need blood transfusion frequently and, when on hemodialysis, they present a 20-50% diminished lifetime of the erythrocytes, also contributing to false values of A1C (43). The increased uremia in CKD results in the production of carbamylated Hb, an in vitro interfering factor in some analytic methodologies for A1C (6).

Some studies have demonstrated that GA provides a more precise control of glycemia in patients with advanced stages of CKD (14,15,42). However, in the presence of massive proteinuria with diminished serum albumin, the GA levels can also be falsely altered (42,44), and it is necessary to perform a critical evaluation and adequately choose the best glycemic marker in this condition.

## GA IN THE DIAGNOSIS OF DM

Despite the evident informative value of A1C in monitoring DM, some authors have questioned the cutoff point used for this test in diagnosing the disease. This is because the current criteria adopted show a discrepancy between the proportion and profile of patients identified as having DM by the A1C, compared to the tests based on glycemia (45,46). In addition, the patients who present special conditions that interfere with A1C results should be screened for DM with alternative markers. Since the enzymatic method for GA was recently developed, few diagnostic accuracy studies of GA for DM have been published (Table 1).

**Table 1:** Diagnostic accuracy studies of GA and the cutoff points found to screening DM

Study	N	Country	Male	RI GA (%)	DM cutoff	SxS
Tominaga and cols. 2006	699	Japan	52%	12.3 - 16.9	-	-
Paroni and cols. 2007	32	Italy	37%	11.7 - 16.9	-	-
Kohzuma and cols. 2011	201	USA	47%	11.9 - 15.8	-	-
Furusyo and cols. 2011	1.575	Japan	30%	12.2 - 16.5	15.5%	83.3 x 83.3
Hwang and cols. 2014	852	Korean	58%	-	14.3%	66.4 x 52.5
Ikezaki and cols. 2015	176	Japan	46%	-	15.2%	62.1 x 61.9
Hsu and cols. 2015	2.192	Taiwan	50%	-	14.9%	78.5 x 80.0
Testa and cols. 2017	252	Italy	38%	9.0 - 16.0	-	-

RI GA: reference interval for GA; SxS: sensitivity and specificity to the cutoff points found.

In 2006, the Japan Diabetes Society (JDS) established a reference interval for GA from 12.3% to 16.9% (47). Years later, in a larger study (N = 1.575), Furusyo and cols. published a reference interval for GA from 12.2% to 16.5%, corroborating with that reported by the JDS. Further, this study found that the cutoff point of GA ≥ 15.5% presented a good sensitivity and specificity (both 83.3%) to identify DM, using FG and/or A1C (≥ 126 mg/dL and ≥ 6.5%, respectively) as reference tests (48). In 2015, the same group evaluated 176 residents of Japan diagnosed with DM by the OGTT, according to WHO criteria. ROC curve analysis showed that GA presented significant differences in the area under the curve (AUC) for DM diagnosis, and these values increased when combining GA with FG or 2hG than GA when isolated (AUC: 0.863, 0.968 and 0.672, respectively) (49).

Hwang and cols. assessed different cutoff points of GA for DM and pre-DM diagnosis in 852 Korean adults, using the ADA criteria to classify the disease. The study reported a cutoff point of 12.5% for pre-DM and 14.3% for DM. GA presented greater sensitivity than A1C (66.4% GA versus 52.5% A1C), but less specificity (88.3% GA versus 95.1% A1C) to predict 2 hG ≥ 200 mg/dL. When the values of 14.3% of GA were associated with FG ≥ 126 mg/dL, higher sensitivity was obtained (77.5%, CI: 72.17–82.0) to diagnose DM (50). Hsu and cols. described a cutoff point of GA ≥ 14.9% for DM (sensitivity: 78.5%; specificity: 80.0%), evaluating 2,192 adult individuals in Taiwan. Also, when the values of 5.7% and 6.5% of A1C were considered, the corresponding GA was 14.5% and 16.5%, respectively (51).

Smaller studies described GA intervals in individuals without DM ranging from 11.9 to 15.8% (N = 201 residents of North Carolina, USA) (31); 10.2 to 16.1% (N = 217 African immigrants in America) (52); 10.5 to 17.5% (N = 44 volunteers of a Canadian study) (33); and 9.0 to 16.0% (N = 252 European persons) (53). In young obese persons aged from 10 to 18 years, the value of GA to diagnose DM was ≥ 12% when 2 hG was used as the reference test, and ≥ 14% when A1C was used as reference diagnostic criterion (54).

## GA IN GLUCOSE MONITORING

**Figure 3:**
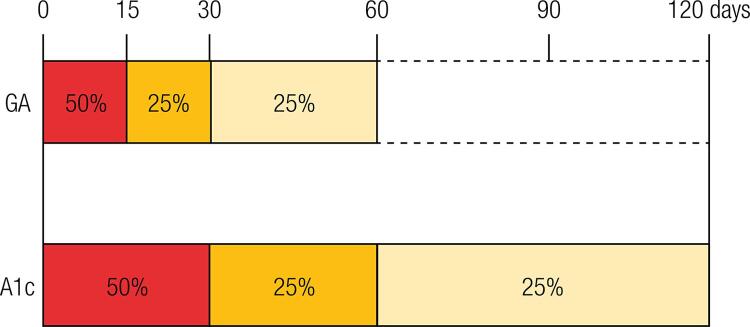
Glycation rates of GA e A1C. GA is produced over the life span of albumin of approximately 8 weeks, however, the first 2 weeks account for half of its production. Differently, due to the life span of erythrocytes, that is around 120 days, A1C takes approximately 4 months to be completely produced, and the first month is responsible for half of its glycation.

Differently from A1C long-term formation (about 120 days, mean life of the erythrocytes), GA is formed in a period of approximately 2 to 4 weeks (Figure 3) (37). This feature enhances GA sensitivity to the rapid alterations in glucose levels, which may not be efficiently identified with an isolated measure of plasma glucose (13,19).

Compared to A1C, GA is more suitable to monitor the beginning of drug therapy in DM (55), and also to control the dose and change of medication (51), since its levels diminish faster than A1C in intensive treatment (19). Paroni and cols. evidenced that GA was a better marker to evaluate the responses to treatment with insulin in type 2 DM patients with inadequate glycemic control, and also that GA presented a greater correlation with FG than A1C (R = 0.75 versus R = 0.54, respectively) (30). Moreover, Yoon and cols. reported that the worsening of the beta-pancreatic cell function was associated with the time of duration of DM, and also with increased GA and GA/A1C ratio, but not with A1C alone (56).

In general, GA can be employed to show mean glycemia and also to evaluate the glycemic variability and postprandial glucose levels more adequately than A1C (37,57). Elevations in postprandial glycemia are associated with the increased risk of cardiovascular diseases and microangiopathy, thus the detection of these glucose variations is important (37). The reasons why GA is better related to postprandial glycemia have not yet been elucidated (11).

## GA AS A PREDICTOR OF LONG TERM COMPLICATION IN DM

The chronic hyperglycemia considerably increases the risk of developing micro and macrovascular diseases over time (1,2). A1C is a marker that has been strongly explored in clinical research and much evidence has supported its use as a predictor for these complications in DM (4,5). However, there is still controversy if mean glycemia itself or glycemic variability is the main determining factor for chronic damage in DM (16). Recent studies have evaluated the predictive potential value of tests that are more associated with the short term glycemia and that can be used as alternative markers for A1C, such as GA (8,16,17,58).

Selvin and cols. cross-sectionally evaluated 1,600 individuals recruited for the Atherosclerosis Risk in Communities (ARIC) study conducted in the USA. They observed that in participants with type 2 DM, both GA and fructosamine were significantly associated with the prevalence of albuminuria, CKD, and retinopathy (8). In a longitudinal study, the same group evaluated 12,306 participants from ARIC, who were followed for over 20 years and demonstrated that both GA and fructosamine were similarly associated with A1C to predict retinopathy and CKD in DM. These findings were confirmed in patients diagnosed with DM during the baseline period and in those who developed DM during the follow-up. The odds ratio (OR) observed for the onset of retinopathy in patients with DM and GA levels between 15.7% and 23.0%, was lower than when GA > 23.0% (OR > 8 and OR > 15, respectively), even in a statistical model adjusted for the A1C levels (17). In addition, Selvin and cols. have also demonstrated a similar association between GA and A1C regarding coronary heart disease, ischemic stroke, heart failure, and death (59).

Nathan and cols. used data from the DCCT and Epidemiology of Diabetes Interventions and Complications (EDIC) studies to evaluate the correlation between GA and chronic complications in type 1 DM. They showed that GA, as well as A1C, were strongly associated with the onset of retinopathy and nephropathy after a mean follow-up time of 6.5 years, but none evidence was seen for 7-points glucose profile. Only A1C was associated with cardiovascular disease (16). In another smaller study with 154 type 1 DM patients followed over 2.8 years, the progression to nephropathy was associated only with GA and not with A1C. The authors did not find association between these two glycemic markers and the cardiovascular outcomes (58).

Apparently, GA is predictive for microvascular complications both in type 1 and type 2 DM. However, regarding macrovascular outcomes, GA seems to be a good marker only in type 2 DM. The mechanisms involved in the development of atherosclerosis and cardiovascular diseases in type 1 DM might explain these findings.

## LIMITATIONS OF GA

Some situations that interfere with albumin metabolism may also influence GA values. Theoretically, GA is not altered by the serum albumin levels, since its values are corrected for the total albumin, but low levels of this protein are associated with increased glycation rates. On the other hand, increased protein metabolism implicates in lower GA levels (60). Therefore, in conditions as hyperthyroidism, hypothyroidism, liver cirrhosis, nephrotic syndrome with massive proteinuria, or other specific disorders, the use of GA may be misleading and should be avoided (32). However, because this test is relatively new, few studies have been carried out to verify interfering factors in GA levels.

Other interfering situations on GA levels already described are age, obesity and inflammatory conditions (observed by the increase of C-reactive protein), smoking, and hypertriglyceridemia (11,32,37). There is little evidence regarding the interpretation of GA in different ethnic groups. However, Selvin and cols. analyzed 1,376 persons without DM and 343 with DM, and found that both GA and A1C are significantly elevated in Blacks compared to Whites (8). Thus, the data presented here show the necessity of being careful when interpreting GA levels in some clinical situations.

## CONCLUSIONS

GA is a short-term marker of glycemia that has been evaluated as an alternative test to A1C in patients with DM. If compared to A1C, GA is more reliable to evaluate glycemic variability. Also, it is especially indicated for patients on hemodialysis and its levels are not affected in the presence of anemias or hemolytic processes. Compared to the fructosamine test, GA is more advantageous, since it is not influenced by other serum proteins. The enzymatic methodology for its analysis is easy and quick to implement, and highly efficient analytically and with greater standardization. As previously described, in clinical situations that falsely alter A1C levels, the measurement of GA may assign a reliable result for monitoring DM. However, the physiology of the formation of these two glycated proteins ensures advantages to GA compared to A1C in access glucose control, even in the absence of interfering factors. Finally, many studies have shown that GA has good diagnostic accuracy and is strongly associated with the diabetic microvascular complications. Despite all benefits of GA, it does not replace the use of A1C, once each test has its advantages and limitations. The choice regarding which test to use should be guided by the clinical patient features and tests availability. Further, it is necessary an international consensus about laboratory issues and clinical use of GA, to guarantee its inclusion in the routine of clinical laboratory worldwide, thus improving the future screening and management of DM patients.
